# Improving Performance of Cluster Heads Selection in DEC Protocol Using K-Means Algorithm for WSN

**DOI:** 10.3390/s24196303

**Published:** 2024-09-29

**Authors:** Abdulla Juwaied, Lidia Jackowska-Strumillo

**Affiliations:** Institute of Applied Computer Science, Lodz University of Technology, Stefanowskiego 18, 90-537 Lodz, Poland; abdulla.juwaied@dokt.p.lodz.pl

**Keywords:** wireless sensor networks, deterministic energy-efficient clustering, K-means, energy consumption, network’s stability period

## Abstract

Wireless sensor networks (WSN) have found more and more applications in remote control and monitoring systems. Energy management in the network is crucial because all nodes in the WSN are energy constrained. Therefore, the design and implementation of WSN protocols that reduce energy depletion in the network is still an open scientific problem. In this paper, we propose a new clustering protocol that combines DEC (deterministic energy-efficient clustering) protocol with K-means clustering, called DEC-KM (deterministic energy-efficient clustering protocol with K-means). DEC is a very energy-efficient clustering protocol that outperforms its predecessors, such as LEACH and SEP. K-means ensures more effective clustering and shorter data transmission distances within the network. The shorter distances improve the network’s lifetime and stability and reduce power consumption. Additional heuristic rules in DEC-KM ensure improved cluster head selection, taking into account node energy level and position and minimising the risk of premature cluster head exhaustion. The simulation results for the DEC-KM protocol using MATLAB show that cluster heads have shorter distances to nodes in cluster areas than for the original DEC protocol. The proposed protocol ensures reduced energy consumption, outperforms the standard DEC, and extends the stability period and lifetime of the network.

## 1. Introduction

Wireless sensor networks (WSN) have been considered one of the most significant technological achievements of recent years. Wireless communication and the Internet are constantly used in medicine, technology, agriculture, environmental protection, etc.

Small, intelligent sensors enable the construction of portable devices for measuring and recording biomedical signals and monitoring the patient’s health outside the medical centre. A newly developing field is textronics, which deals with the design of clothing with built-in microsensors that monitor various parameters of the health or well-being of drivers, firefighters and other people performing dangerous work or responsible for the health of others.

In control and measurement systems, WSN and small smart sensors are irreplaceable in hard-to-reach places, or they can be deployed in a physical area on an inaccessible track [1,2,3].

**Figure 1:**
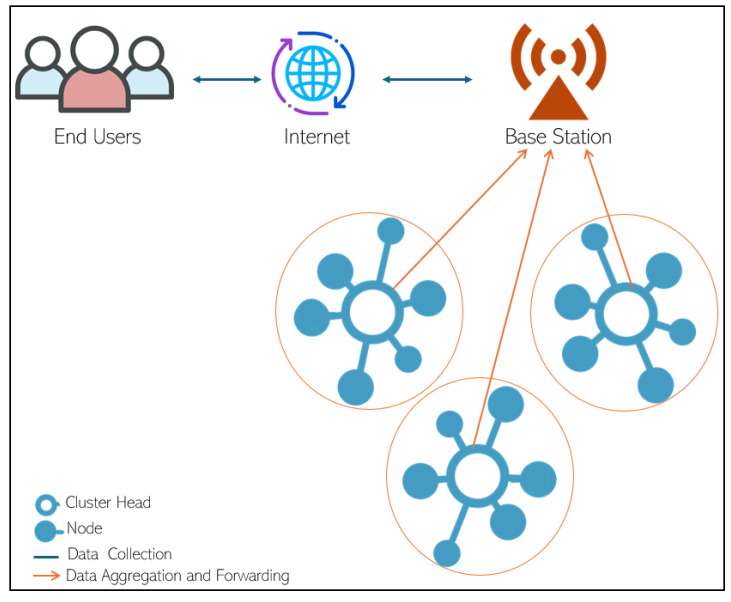
Clustering in hierarchal routing protocol.

All WSNs consist of a base station (BS) connected to the Internet and a set of sensor nodes, which communicate with the base station by a wireless channel (see Figure 1). Sensors are small and complex devices; they can sense, process and transmit the data to other sensors in the same network. Therefore, all nodes consist of four main parts: the power part, which is responsible for battery level and energy consumption of sensors; the communication part, used to send and receive data in the network; the processing part, accountable for processing the data and the sensing part used to read data from neighbour nodes [2].

All nodes in WSN are energy constrained. Therefore, in recent years, researchers have developed and implemented many WSN protocols that decrease the energy depletion in the network. [3].

Wireless sensor network protocols can be classified into three main types depending on the network architecture: hierarchical routing protocols, flat routing protocols, and location routing protocols. The most popular are hierarchal routing protocols. In this protocol type, the network is divided into smaller groups called cluster areas. Every cluster area consists of only one cluster head (CH) and a set of nodes. All cluster heads have the same functions: connect nodes in the same cluster area to collect data, process the data, and send the data to the base station of the network [4], as shown in Figure 1.

The most commonly used hierarchal routing protocols are LEACH (low-energy adaptive clustering hierarchy) [2,5], SEP (stable election protocol) [6], TEEN (threshold sensitive energy efficient sensor network) [4], DEEC (distributed energy-efficient clustering) [7], DEC (deterministic energy-efficient clustering) [1,8] and their modifications [5,9].

In LEACH [10], cluster heads in the network are selected randomly, and it does not consider the nodes’ location in the same cluster area. Because of that, many cluster heads will be near each other or near the edge of the network, leading to higher energy consumption. In SEP [6], the cluster head selection is made randomly, similar to the LEACH protocol, but there are two different types of nodes: normal nodes and advanced nodes, which allows for taking into account the network heterogeneity. In TEEN [11], cluster heads are also selected randomly, and energy consumption is reduced by introducing thresholds, reducing the number of transmissions by allowing the nodes to transmit only when the sensed attribute is in the range of interest. One of the prevalent drawbacks of this protocol is when the hard threshold is not checked correctly. Then, the nodes in the cluster area will not connect, and the user will not obtain information about the dead cluster head in the network. In DEEC, the cluster heads are elected by probability based on the ratio between the residual energy of each node and the average energy of the network. In DEC, the CH election is locally decided based on each node’s residual energy (RE). DEEC and DEC are dedicated to heterogeneous environments and improve the lifetime of wireless sensor networks significantly when compared with LEACH and SEP [1,7].

In our previous works, we presented modifications of LEACH [12], SEP [13] and TEEN [14] protocols, leading to a better selection of cluster heads by using a K-nearest neighbour algorithm for minimising energy consumption.

In this paper, we propose a new clustering protocol that combines the DEC protocol with K-means clustering, which improves the performance of cluster head selection, which is called DEC-KM (deterministic energy-efficient clustering protocol with K-means).

K-means stands out as a widely utilised clustering algorithm in machine learning and data mining due to its ability to effectively balance speed and accuracy [15]. K-means is the preferred choice because of its speed, as it is efficient in computation and implementation, making it well-suited for handling large datasets. Additionally, it is accurate and produces reliable, well-defined clusters, particularly for spherical data distributions. In wireless sensor networks (WSNs), K-means clustering is highly beneficial compared with random clusterisation for several reasons. Its capacity to enhance network performance, increase energy efficiency, and simplify data aggregation are key factors contributing to its effectiveness in WSNs.

All cluster heads will have a better position in the network because the distance between nodes in each cluster area will be calculated and compared between other nodes to find the shortest using the K-means algorithm.

Additionally, the set of heuristic rules is proposed in the last algorithm stage, which ensures choosing the nodes of high energy level close to the centre point in a cluster area and close to the base station, as well as rotating the CHs whose energy has been exhausted due to data transmission.

## 2. Related Work

The deterministic energy-efficient clustering (DEC) protocol is dynamic, distributive, self-organising and a very energy-efficient cluster protocol [1]. It outperforms LEACH and SEP protocols because the CH election is not random but determined by each node’s residual energy [16]. Popular solutions are sensing applications, which evaluate nodes’ energy based on the strength of their response, increase the sensor lifetime and network security, and decrease power consumption and cost [3].

In recent years, improving the DEC protocol has been the goal of many research efforts. Energy efficient multitier random DEC protocol [17] is a new standard DEC protocol to enhance the network’s work lifetime and energy efficiency. The protocol incorporates a novel normal random initialisation and a multitier structure. Additionally, it aims to reduce the uncertainties associated with the cluster head elections process. The research reveals that the modifications effectively ensure a uniform selection of cluster heads. This enhancement results in improved energy efficiency for the protocol, owing to its superior distribution capacity.

An efficient and ideal modified multitier deterministic energy-efficient clustering protocol, based on a novel election multitier random probability approach in a modified non-residual format called NR-MDEC, has been introduced in [18]. This protocol can maximise utilising the network’s critical resources regarding residual energy level after 60% of CHs is dead. In general, and in the other conditions, DEC outperforms NR-MDEC.

The weaknesses of DEC include its failure to consider the intra-cluster distance and the degree of the node. Particle Swarm Optimisation (PSO) based deterministic energy-efficient clustering protocol, PSO-DEC, was introduced to overcome these challenges [19]. MATLAB-based recreation output showed that PSO-DEC outwits conventional DEC and improves the network’s lifetime. The weakness of Particle Swarm Optimisation is the long calculation time, which could be unacceptable for real-time online applications.

Distance-based deterministic energy efficient clustering protocol (DDEC) [20] outperforms probabilistic protocols by selecting a uniform and well-distributed number of CHs throughout the sensor field. Simulations showed that DDEC has a more stable region period than ESEP, DEC, and LEACH protocols [21]. The network’s stability period in DDEC is 12.7% longer than for the original DEC protocol.

A distributed energy-efficient clustering protocol called DCE for heterogeneous wireless sensor networks introduces an extra phase for cluster head election [9]. DCE outperforms DEEC, SEP and LEACH algorithms in terms of stability period.

The proper clustering algorithm is key to reducing energy consumption and increasing the network lifetime. Therefore, the design of new clustering algorithms is still a scientific challenge.

In this work, we propose a new modification of the DEC protocol based on the K-means algorithm. The K-means algorithm has already been implemented by other researchers for WSN clustering. However, to the authors’ knowledge, no attempt has been made to combine DEC and K-means.

Most researchers implemented the K-means clustering combined with the LEACH protocol [22,23,24] or its modifications. In [22], optimal K-means clustering combined with LEACH protocol is used and compared with LEACH, O-LEACH and the BeeCluster, which is a static clustering routing algorithm based on an artificial Bee colony. The proposed algorithm achieved a uniform spatial distribution of CHs and 28% lesser energy consumption as compared to its contender. A new KCED protocol [23,24] combining K-means and LEACH has been proposed to address the issue of residual energy within a node, cluster centre, and distance to a base station.

A two-dimensional wireless sensor network (WSN) model was utilised to generate different WSN designs to optimise them using genetic algorithms to attain the best-performing WSN models [25]. The distance measure was utilised to identify the handled problem, and K-means clustering was employed to reposition sensors around the alternative cluster head

Fuzzy-based hybrid clustering and K-means algorithm for wireless sensor networks were proposed to resolve the problem of load balancing and cluster head (CH) selection in cluster areas with minimum energy expenditure [26]. This technique proposes a hybrid methodology that uses an unsupervised machine learning-based K-means algorithm with a fuzzy logic approach to choose cluster heads. More parameters and factors can be considered to improve this technique, such as the distance between nodes and the location of CH to BS.

The DCK-LEACH protocol [27] is based on K-means and Canopy optimisation. This research depends on combining the dynamic Canopy algorithm and the K-means algorithm for effective clustering. This study presents a method for managing the frequency of reclustering processes to decrease energy waste. This algorithm utilises a hierarchical approach for the election of cluster heads, which alleviates the workload of the cluster head and achieves a balanced distribution of network traffic.

A study [28] proposes using an unmanned aerial vehicle (UAV) as a flying relay to receive and transfer signals using nonorthogonal multiple access (NOMA) for optimal spectrum-sharing efficiency by using the K-means unsupervised machine learning and the gap statistic approach to cluster sensors and optimise UAV location. This work presents a method to optimise UAV trajectory: the centroid-to-next-nearest-centroid (CNNC) route. The study found that the K-means algorithm is effective for deploying large and complex WSNs.

## 3. DEC (Deterministic Energy-Efficient Clustering) Protocol

### 3.1. DEC Characteristics

**Figure 2:**
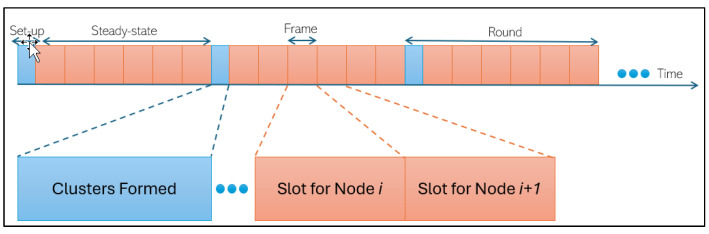
DEC protocol operation: in the set-up clusters are formed and in the steady-state frames are transmitted.

The deterministic energy-efficient clustering (DEC) protocol is a clustering protocol designed for wireless sensor networks (WSNs) to improve energy efficiency and prolong the network’s lifetime. It focuses on organising nodes into clusters and efficiently managing the communication and data transmission within these clusters. DEC aims to reduce energy consumption, minimise data transmission overhead, and extend the operational lifespan of battery-powered sensor nodes [19,20]. The DEC protocol divides the network into several rounds, and every round contains two stages: the set-up and steady-state stages, as shown in Figure 2. The DEC protocol has three main operations in the set-up stage: start to select cluster heads, choose cluster heads randomly, and establish. Data transmission will be completed in the steady-state stage, and the information will be transferred from cluster heads to a base station [18,21].

The set-up stage in the DEC protocol is different from other protocols. This stage is divided into three main parts, as mentioned earlier. When the number of rounds increases in DEC protocol, some problems appear that affect the network’s performance. For example, the nodes become dead earlier than in LEACH, SEP and TEEN. Therefore, balancing the stability and number of rounds in the network is challenging [1,17].

The cluster heads and node sensors will be selected in DEC protocol depending on the remaining energy level, which can be calculated depending on the threshold value from Equation (1) [29]. The selection is made by comparing the remaining energy of each node with neighbour nodes [16,30].

(1) $$T(n_{x})=\left\{\begin{array}{cc} P_{x}/(1-P_{x}\;(r\;mod\;1/P_{x})) & Q\;if\;n_{x}|\;\epsilon \;G\;\\ 0 & otherwise, \end{array}\right.$$

where: $T(n_{x})$—threshold calculated depending on which sensor is selected as cluster head in each round r, r—round, $P_{x}$—the probability of change in node to become a CH, G—is the set of nodes that will be chosen as CHs at each round r, Q—is a parameter of the remaining energy of each node.

### 3.2. DEC Energy Dissipation and Data Aggregation Model

**Figure 3:**
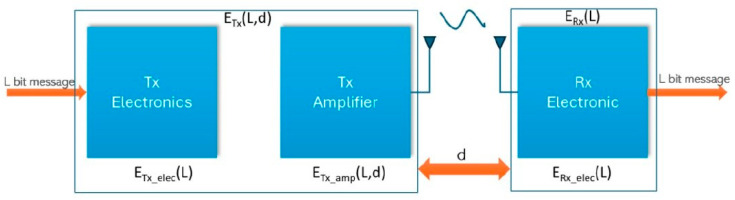
Energy network model diagram for the single x node.

The DEC protocol is analysed depending on the energy dissipation and data aggregation model [20,31] shown in Figure 3. The transmission of the *L* data bits over a distance (*d*) is presented. The energy of nodes is lost when receiving and transmitting data in the network. The energy dissipation model consists of three parts: the radio electronics, the power amplifier transmitter, and the radio electronics receiver. The amplification energy E will be used to overcome free space (*fs*) or multipath (*mp*) loss.

The transmitted energy *E_Tx_* of the size of a packet *L-bit* message over the distance of transmitted data (*d*) can be calculated from the following equation [14]:

(2) $$E_{Tx}=\left\{\begin{array}{c} L\;E_{elec}+t\;E_{fs}d^{2}\;if\;d<d_{0}\\ L\;E_{elec}+t\;E_{mp}d^{4}\;if\;d>d_{0} \end{array}\right.$$

where: $E_{Tx}$—is the energy consumption in the sending process, $E_{elec}$—is the energy dissipation per bit in the electronics circuit, L—is the size of the packet (number of bits), $E_{fs}\;{,E}_{mp}$—are the energy dissipation for free space (*fs*) and multipath loss (*mp*), features of *d*, *d*—distance of transmission between two communicating endpoints, *d*_0_—is the distance threshold, which can be calculated from Equation (3):

(3) $$d_{0=}\sqrt{\frac{E_{fs}}{E_{mp}}}$$

$E_{Rx}$—is the energy consumption in the receiving process, which can be calculated from Equation (4):

(4) $$E_{Rx\;}=E_{elec}\;L$$

## 4. DEC-KM (Deterministic Energy-Efficient Clustering Protocol with K-Means)

DEC relies on frequent communication among nodes to coordinate cluster head rotation and other activities. This communication overhead can lead to increased energy consumption, especially in scenarios with high data traffic. As nodes start to choose CHs randomly in most scenarios, the DEC may not achieve perfect load balancing among nodes, leading to some nodes still experiencing higher energy depletion rates than others. Regarding the network topology and deployment, the DEC may still face the risk of premature cluster head exhaustion, where some nodes deplete their energy faster than others. While the rotation mechanism helps, it may not eliminate this issue. To modify the selection approach of cluster head in the DEC protocol, we propose using the K-means algorithm, an essential flat clustering algorithm.

K-means clustering is based on centroids and minimises the variance within each cluster, resulting in clearly defined and compact clusters. K-means is particularly effective for spherical clusters, as it can accurately group data that naturally forms into such clusters. With deterministic convergence, K-means will consistently yield the same results when given the same initial centroids, ensuring reliability and repeatability. Additionally, it is versatile, performs well with different data types, and can be adjusted with various distance metrics to enhance accuracy for specific datasets [15].

The k-means is an iterative algorithm. It chooses the centroids depending on the position of other nodes, which can be defined as a centre point between all nodes in the same cluster area. The algorithm result depends on the choice of the initial centre points. To obtain a low level of energy consumption and improve the lifetime and stability of the network, we propose to modify the DEC protocol through three main stages:

- STAGE 1: Calculate the initial cluster areas and cluster heads using DEC protocol.
- STAGE 2: Apply the K-means algorithm for the data calculated in STAGE 1 and make a new clustering. This stage can be divided into three sub-steps:
  - Step 1 Centro: Calculate the new centre point of each cluster area in the network from Equation (5):
    $$\mu_{i}\left(\omega_{i}\right)=\frac{1}{\left|\omega_{i}\right|}\sum_{x\in \omega_{i}}^{n}x$$
    The results will be stored as *x* and *y* coordinates. The centroids will be used as a reference to choose the new cluster head for the nodes.
  - Step 2 Node Distance: The distances to the centre points will be calculated for every node, and the node will be assigned to the closest centre point. The metrics used to calculate the distances of the node to the centroids are given by Equation (6):
    $$D=\sum_{x\in \omega_{n}}^{n}{\left|x-\mu (\omega_{n})\right|}^{2}$$
  - Step 3 Stop condition: If there are no changes in clusters or a maximum number of iterations is reached, the clustering is finished; if not, return to *Cento* and repeat until the stop condition is not fulfilled.

- STAGE 3: CH Selection:
  - Step 1: For each CH calculated in Stage 1 using DEC protocol, check if it still belongs to the same cluster area and if its distance to the Base Station (BS) is shorter than the distance of cluster centroid to the BS:
    – If Yes: return the CH as a confirmed CH.
    – If No: Using the coordinates of centre points and distances, which were calculated in Stage 2, compare node distances to the centroid in the same cluster area and find the node nearest to the centroid. The node with the shortest length to the centroid point will be chosen as a tentative CH of this area.
  - Step 2: For each tentative CH, calculate the energy threshold needed to transmit and receive data using the DEC energy model. Check if the CH energy level is over this threshold:
    – If Yes: return the new CH as a confirmed CH.
    – If No: choose the next tentative CH nearest to the centroid point and return to Step 2. If there are no more nodes in the cluster area, eliminate the cluster with the node energy below the given threshold and assign its nodes to the closest cluster heads.

## 5. Simulation and Evaluation of DEC and DEC-KM Protocols

In this chapter, simulations conducted for the DEC and the modified DEC-KM protocol with K-means have been presented and compared. Both simulations were performed for the same parameters, as shown in Table 1.

### 5.1. DEC Protocol Implementation

The simulation and analysis for the original DEC and DEC-KM protocols were performed using MATLAB for the same parameters and initial conditions. The simulation environment involves three sets of nodes containing 50, 100 and 200 nodes, initially randomly distributed in the 100 m × 100 m field. The network is heterogeneous, and the nodes have different energy levels. The Base Station (BS) is located in the centre of the network. The nodes were organised into 10 clusters. All simulations were performed for 50 nodes, and only the simulations presented in Section 5.2.5. in Figures 10 and 11 and in Table 6 were carried out for three networks consisting of 50, 100 and 200 nodes.

**Figure 4:**
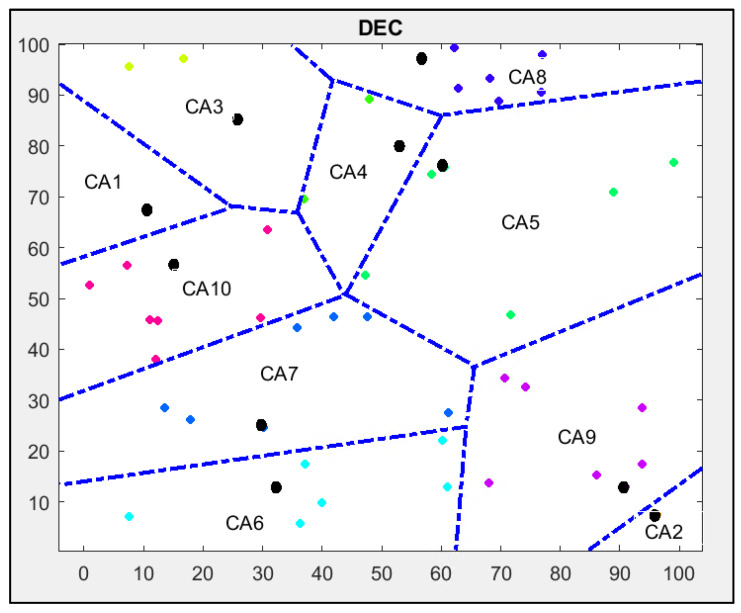
Results of the implementation of standard DEC protocol using MATLAB: cluster heads are marked as bold black circles, and other nodes are marked as colour rhombus; nodes in the same cluster area are marked with the same colour.

The other parameters used for all simulations are shown in Table 1. The results obtained for the DEC protocol are shown in Figure 4.

The network contains a set of nodes of different energy levels that are uniformly randomly sorted and divided into 10 clusters. All nodes communicate with the base station located in the centre of the network via cluster heads marked as black circles.

In connection and processing, all nodes have similar capabilities, but their energy levels are different because the level of energy consumption is different from node to node; therefore, some nodes, for example, CH, need more energy than the normal nodes.

The clustering of the network shown in Figure 4 has its drawbacks. There are long distances between the nodes in some cluster areas, for example, in CA5, CA6 and CA7. Two clusters (CA1 and CA2) contain only one node.

### 5.2. Simulation and Evaluation of DEC-KM

The simulation for the modified DEC-KM protocol has been conducted for the same initial conditions and parameters described in Section 5.1 and gathered in Table 1.

#### 5.2.1. Algorithm Implementation

The protocol has been implemented in three main stages:

STAGE 1: Using the parameters from Table 1, implement the network and calculate the initial cluster areas and cluster heads using the DEC protocol.

STAGE 2: Apply the K-means algorithm for the data calculated in STAGE 1 and make a new clustering.

STAGE 3: Perform CH Selection, checking if the CH energy is over the threshold; choose the nodes of high energy level close to the centre point in a cluster area and the base station.

#### 5.2.2. Choosing Centroid Points for K-Means

In the first step in Stage 2 of the DEC-KM algorithm, the K-means centroids are calculated in every cluster area obtained from DEC protocol and shown in Figure 4.

**Figure 5:**
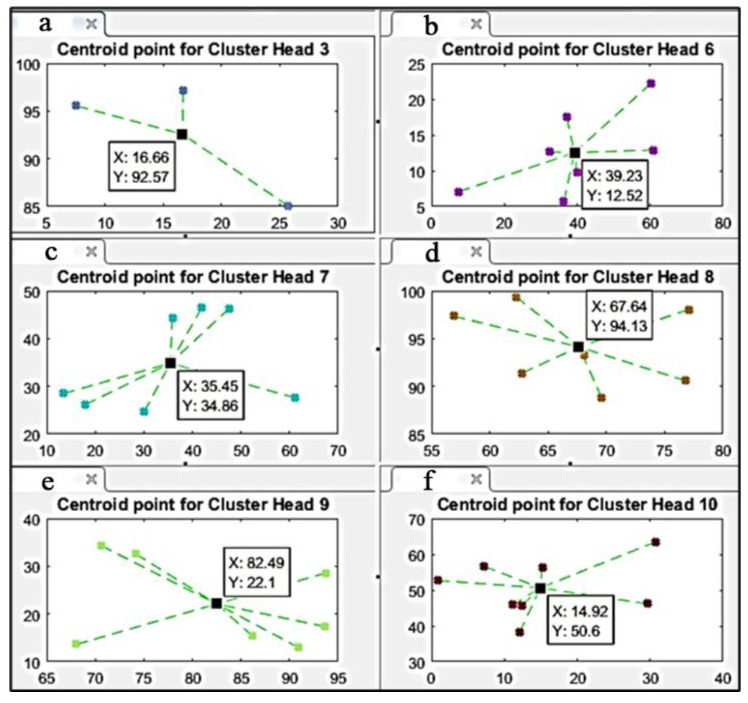
Centroid points for selected cluster areas in the first K-means iteration.

Centroid points for selected cluster areas after the first K-means iteration are shown in Figure 5. The black square in each cluster area marks the centroid point of this area.

#### 5.2.3. Simulation Results

**Figure 6:**
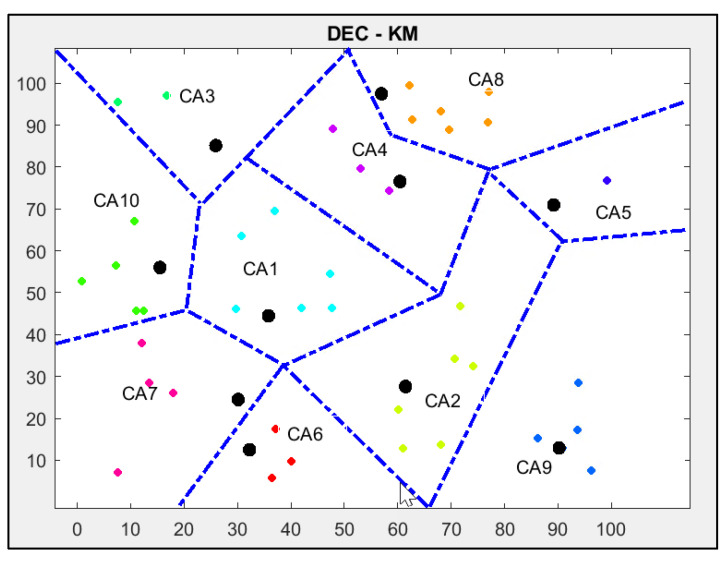
Results of the implementation of standard DEC-KM protocol: cluster heads are marked as bold black circles, and other nodes are marked as colour rhombus; nodes in the same cluster area are marked with the same colour.

The results obtained for the DEC-KM protocol are shown in Figure 6.

Comparing the simulation results in Figure 4 and Figure 6, we see that network clustering is more effective for the DEC-KM algorithm. Distances between the nodes inside the cluster area are shorter than for the DEC protocol, and no single-node clusters exist.

After applying the DEC-KM algorithm using the same parameters that implemented the original protocol, six cluster heads (in cluster areas CA3, CA6, CA7, CA8, CA9, CA10) remained in the same place as for the DEC protocol. However, some cluster areas have been changed. Cluster heads in CA1, CA2, CA4 and CA5 areas contain the newly chosen cluster heads depending on centroid points in the cluster areas and their energy level.

#### 5.2.4. Comparison of Clusters and Link Distances between CHs for DEC and DEC-KM

**Figure 7:**
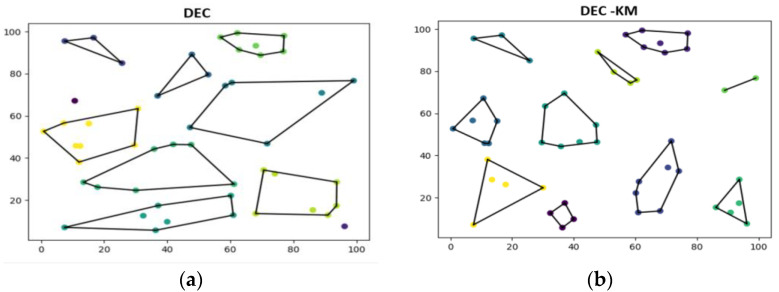
Network clustering and node communication in the network: (**a**) in original DEC and (**b**) in DEC-KM; nodes in the same cluster area are marked with the same colour.

A comparison of both protocols was performed using computer simulations. Figure 7 shows clustering results for the DEC and the DEC-KM. It is well seen that the DEC-KM protocol divides the network into more compact clusters. Therefore, the link distances between nodes in the cluster are less than for the original protocol. Comparisons of changes in clusters and the link distances are shown in Table 2.

Results gathered in Table 2 indicate that after K-means clusterisation, two clusters and cluster heads (CH3 and CH8) remained unchanged; five cluster heads (CH5, CH6, CH7, CH9, CH10) remained in the same place, but their clusters have changed, and three new clusters have been created with new cluster heads (CH1, CH2, CH4).

The sums of the distances and average distances from the cluster head to the nodes for the original and the modified protocol have been computed and compared in Table 2 for the cluster heads, which remained in the same places, but their clusters have changed. In all cases, K-means clustering improved the network structure. Link distances from the nodes to their cluster heads decreased significantly. The sums of the distances in the cluster areas for the original DEC protocol are less than for the DEC-KM in the 40% to 80% range.

Comparisons of the nodes’ shortest and longest link distances to their CH are shown in Table 3, and the total and average longest distances are in Table 4. Results in Table 3 and Table 4 are related only to the five cluster heads determined from the DEC protocol, which remained in the same place, but their clusters have changed. The node’s shortest distances to CH are the same for both protocols, but the sum of the node’s most extended distances in DEC-KM is 42% shorter than in the original DEC protocol.

As seen in Table 5, the sum of CHs’ distances to the base station in DEC-KM did not increase; it is 12% shorter than in the original DEC protocol.

#### 5.2.5. Energy Consumption Analysis

**Figure 8:**
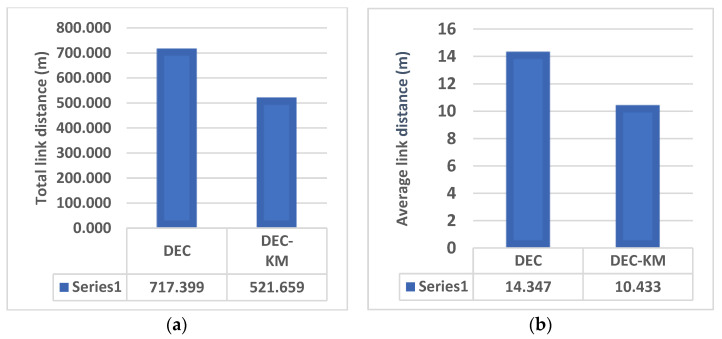
DEC and DEC-KM: (**a**) total link distances between sensors in the network, and (**b**) average link distance per node.

**Figure 9:**
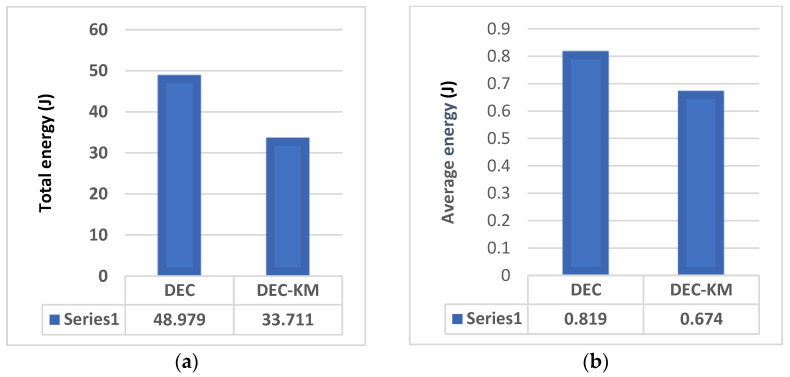
DEC and DEC-KM: (**a**) energy consumption of the network, and (**b**) average energy consumption per node.

When dealing with power management in wireless sensor networks or similar systems, the issue of energy consumption and “dead nodes” or nodes running out of power is a critical concern. Dead nodes can disrupt network operations and reduce the system’s overall effectiveness. As shown in the previous part, the total and average link distances for DEC-KM were less than for the original DEC protocol. Therefore, CH’s average energy consumption and the network’s whole energy consumption will be less than that of the original protocol. Comparisons of the total link distances between sensors and average link distance per node for DEC and DEC-KM protocols are shown in Figure 8. Energy consumption in the network and average energy consumption per node are shown in Figure 9.

**Figure 10:**
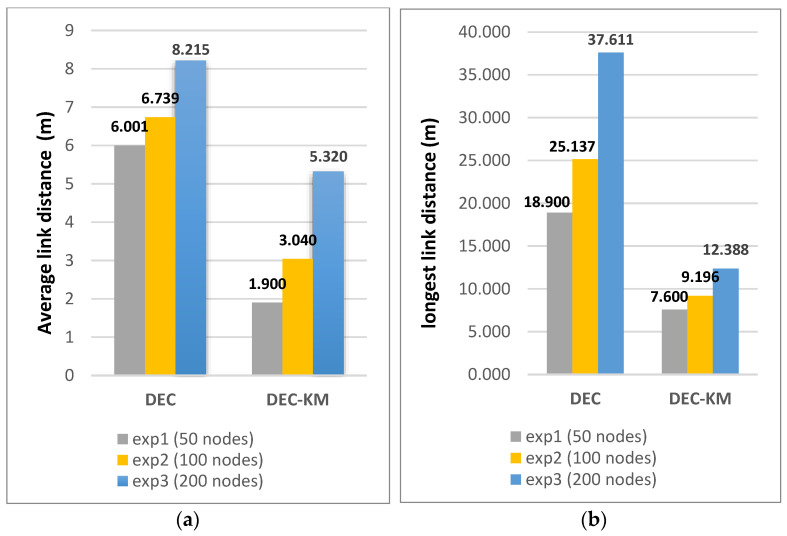
(**a**) The average node link distance in DEC and DEC-KM. (**b**) The longest node link distance in DEC and DEC-KM.

**Figure 11:**
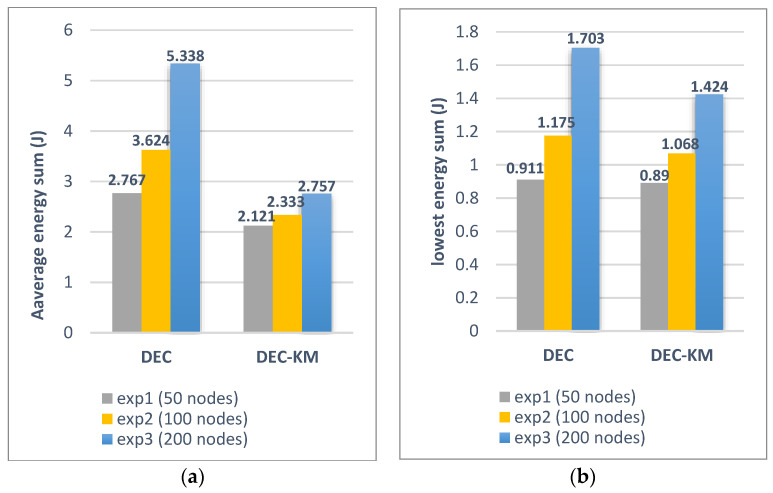
(**a**) The average energy sum per cluster in DEC and DEC-KM. (**b**) The lowest energy sum per cluster in DEC and DEC-KM.

It was also calculated that the sum of the distances between new cluster heads and nodes in the network for DEC-KM is less by 28% than for the original DEC protocol, and the network’s total energy consumption is less by 31%. More detailed calculations of link distances between sensors and energy consumption within the network for DEC and DEC-KM protocols have been carried out by simulation for three networks consisting of 50, 100 and 200 nodes and for parameters given in Table 1. Comparisons of these experiments are presented in Figure 10 and Figure 11 and in Table 6. The average node link distances per cluster and the longest node link distances in the network are shown in Figure 10, and the average energy sum per cluster and the lowest energy sum in a cluster are shown in Figure 11.

The results indicate that the proposed algorithm outperforms the old ones. A summary of improvements of DEC-KM to DEC protocol is gathered in Table 6.

#### 5.2.6. Network’s Stability and Lifetime

Evaluation of the network’s stability and lifetime was performed on the basis of computer simulations conducted for both protocols for 5000 rounds and parameters given in Table 1 and the base station located in the middle of the field.

The following performance metrics were considered:

- FSD—number of rounds until a first sensor node dies—this parameter defines the network’s stability period [17];
- LSD—number of rounds until the last sensor node dies—the period between FSD And LSD defines the network’s instability period [17];
- PSA—number of rounds until 90% of sensor nodes are still alive;
- Chart of the number of alive sensor nodes in a round, i.e., the whole number of sensor nodes whose energy is greater than zero;
- Chart of the number of dead nodes in a round.

**Figure 12:**
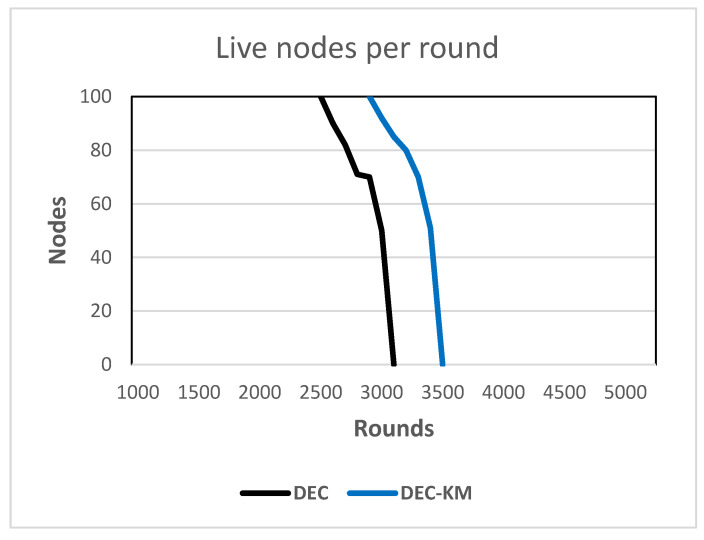
Live nodes per round in DEC and DEC-KM.

**Figure 13:**
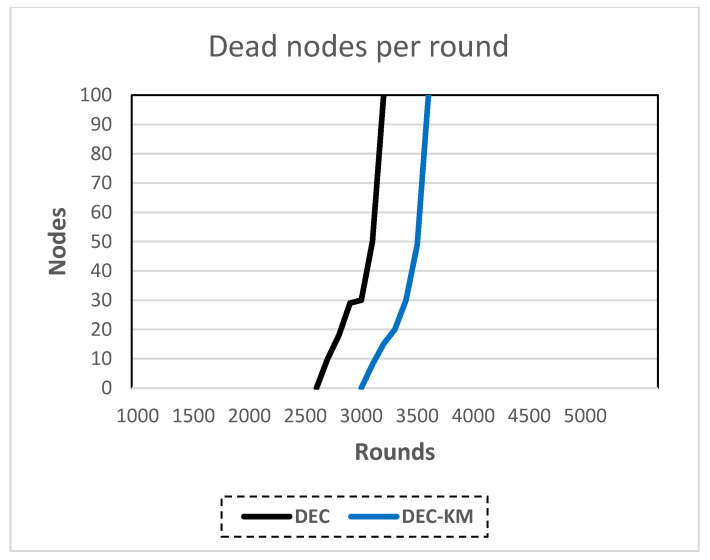
Dead nodes per round in DEC and DEC-KM.

The results of the simulations are shown in Table 7 and in Figure 12 and Figure 13.

The obtained results indicated that the DEC-KM ensures reduced energy consumption, outwits standard DEC and extends the network’s stability period and the network’s lifetime.

## 6. Discussion

Energy management is essential for the Wireless Sensor Network, influencing network security and maximising performance. In this paper, we proposed a heuristic modification of the DEC protocol to decrease and better spread energy consumption in the network. The DEC protocol is a very energy-efficient cluster protocol that outperforms LEACH and SEP protocols because the CH selection is not random but determined by each node’s residual energy. The K-means algorithm is proposed to improve the nodes’ clusterisation and cluster head selection. It was shown that this algorithm chooses the best location of CH depending on the position of all nodes in the same cluster and checks if each CH has the demanded energy level. The set of rules implemented in the last algorithm stage ensures choosing the nodes of high energy level close to the centre point in a cluster area and close to the base station, as well as rotating the CHs whose energy has been exhausted due to data transmission. The DEC-KM protocol takes advantage of the DEC protocol and K-means clusterisation in CH selection.

The computer simulation results showed that by applying the DEC-KM, the sum of the distances between new cluster heads and nodes in the network was less by 28% than in the original DEC protocol, and the total energy consumption was reduced by 31%. The most significant improvement of the proposed solution is more compact clusters, resulting in a reduction of long link distances in the network, even from 40% to 64%. Simulations have been carried out for three different networks consisting of 50, 100 and 200 nodes, and the above-mentioned relationships regarding the reduction of link distances and energy consumption in the networks were confirmed. The new approach also significantly extended the network’s stability period and the network’s lifetime. All performance metrics shown in Table 7 for the DEC-KM protocol are 17.5% to 12% better than for the original DEC protocol, and the network’s lifetime is longer by about 500 rounds.

Comparison with related works [17] indicates that DEC-KM also has a longer stability period than another distance-based DEC protocol (DDEC) without K-means clusterisation. The FSD metric for DEC-KM protocol is 17.5% higher than for DEC, while the FSD for DDEC is 12.7% higher than for the original DEC.

All this determines that the proposed algorithm outperforms the old ones.

## 7. Conclusions

A modified heuristic of the DEC protocol, named DEC-KM, proposed in this paper optimises energy consumption by leveraging the K-means algorithm for improved node clustering and cluster head (CH) selection. The algorithm identifies optimal CH locations based on energy levels and node positions, ensuring efficient energy distribution. Simulations demonstrated a 28% reduction in distances between CHs and nodes and 31% less total energy consumption, with significant decreases in most extended link distances by up to 67%. DEC-KM also prolonged network stability and lifetime, outperforming previous protocols in various performance metrics.

Effective power management in networks with limited energy resources requires a combination of hardware optimisations, efficient algorithms, and network-level strategies. The specific techniques to employ will depend on the characteristics and requirements of the network in question. The proposed DEC-KM algorithm will be a very functional tool that will decrease energy consumption in the network.

Besides the proposed solution, some general strategies used in various networking protocols can be applied additionally to decrease energy usage by implementing power management techniques such as reducing the power state of network interfaces during periods of inactivity. This can include transitioning devices to low-power sleep modes or turning off components that are not currently needed.

Following further enhancements to the DEC-KM protocol, this protocol can be used to communicate with devices, enabling them to communicate and share data, thereby improving automation and operational efficiency. The main application could be in the security and surveillance environment to monitor and detect unauthorised access or movement. The DEC-KM could help to identify changes in motion or voice by providing timely alerts to the base station of potential breaches.

This research has improved the lifetime of the network by minimising the value required to ensure connectivity for all sensors, resulting in lower power consumption, which means a lower cost to build such systems. For future plans, the clustering method should work effectively with the real WSNs, and more factors and parameters can be taken into account to improve the lifetime of the network.

## Figures and Tables

**Table 1:** Parameters used to implement the DEC and DEC-KM protocols in MATLAB.

Parameter	Values	Description
*x × y*	100 m × 100 m	Area of network, dimensions
*n*	50	Number of nodes in the network
*R_max_*	5000	Maximum number of rounds
*P_opt_*	0.1	The probability of a node to become CH
*E_elec_*	50 nJ/bit	Energy dissipation per bit
*E_fs_*	10 pJ/bit/m^2^	Energy dissipation for free space
*E_mp_*	0.0013 pJ/bit/m^4^	Energy dissipation for multipath delay
*E_Rx_*	50 nJ/bit	Receiving energy of sensor
*E_D_*	5 nJ/bit/message	Data aggregation energy
*P_x_*	0.1	Probability of a node to become cluster head
*L*	4000 bits	Packet size

**Table 2:** Comparison of clusters and link distances to cluster heads for DEC and DEC-KM.

	Link Distances of Nodes to Cluster Head in Each Cluster Area				
CH	Sum in DEC (m)	Sum in DEC-KM (m)	AVE in DEC, (m)	AVE in DEC-KM, (m)	DEC-KM DIS. Less DEC, %
**CH1**	New cluster and new CH are obtained after K-means clusterisation				
**CH2**	New cluster and new CH are obtained after K-means clusterisation				
**CH3**	The cluster and CH have not been changed				
**CH4**	New cluster and new CH are obtained after K-means clusterisation				
**CH5**	126.19	29.30	25.23	9.76	77%
**CH6**	106.22	22.79	17.70	7.59	80%
**CH7**	133.64	80.32	22.27	20.08	40%
**CH8**	The cluster and CH have not been changed				
**CH9**	104.67	33.97	17.44	8.49	69%
**CH10**	98.30	56.73	14.04	11.34	43%

**Table 3:** Comparison of the nodes’ shortest and longest link distances to CH for DEC and DEC-KM.

CH	Node Shortest Distance (m)		Node Longest Distance (m)	
	DEC	DEC-KM	DEC	DEC-KM
CH5	2.524	2.524	38.619	18.410
CH6	6.731	6.731	29.357	8.120
CH7	12.232	12.232	31.256	28.614
CH9	5.180	5.180	29.499	15.924
CH10	7.954	7.954	17.691	14.775

**Table 4:** Comparison of the total and average longest link distances for DEC and DEC-KM after 5000 rounds.

Protocol	SUM of the Longest Distances (m)	AVE Long Distance (m)
DEC-KM	85.85	17.17
DEC	146.42	29.28

**Table 5:** Comparison of CHs’ distances to the Base Station for DEC and DEC-KM.

Protocol Sum of CHs’ Distances to Base Station (m)	
DEC-KM	367.08
DEC	417.88

**Table 6:** Improvement of DEC-KM to DEC for 50, 100 and 200 nodes in the network.

Improvement of DEC-KM to DEC	50 Nodes	100 Nodes	200 Nodes
Average energy sum per cluster	Less 23%	Less 36.2%	Less 48.4%
The lowest energy sum in a cluster	Less 2.2%	Less 9%	Less 16.4%
The average node link distance	Less 31%	Less 44%	Less 51.6%
Longest node link distance	Less 40%	Less 64%	Less 67.1%

**Table 7:** Performance metrics of network’s stability and lifetime.

Protocol	FSD	PSA	LSD
DEC	2554	2587	3200
DEC-KM	3002	3011	3590
Improvement of DEC-KM to DEC	17.5%	16.4%	12.2%

## Data Availability

The data is not available because it is used for a PhD thesis in progress.
